# Structural and Functional Diversity of the Microbial Kinome

**DOI:** 10.1371/journal.pbio.0050017

**Published:** 2007-03-13

**Authors:** Natarajan Kannan, Susan S Taylor, Yufeng Zhai, J. Craig Venter, Gerard Manning

**Affiliations:** 1 Department of Chemistry and Biochemistry, University of California San Diego, La Jolla, California, United States of America; 2 Howard Hughes Medical Institute, University of California San Diego, La Jolla, California, United States of America; 3 Razavi-Newman Center for Bioinformatics, Salk Institute for Biological Studies, La Jolla, California, United States of America; 4 J. Craig Venter Institute, Rockville, Maryland, United States of America; Samuel Lunenfeld Research Institute, Canada

## Abstract

The eukaryotic protein kinase (ePK) domain mediates the majority of signaling and coordination of complex events in eukaryotes. By contrast, most bacterial signaling is thought to occur through structurally unrelated histidine kinases, though some ePK-like kinases (ELKs) and small molecule kinases are known in bacteria. Our analysis of the Global Ocean Sampling (GOS) dataset reveals that ELKs are as prevalent as histidine kinases and may play an equally important role in prokaryotic behavior. By combining GOS and public databases, we show that the ePK is just one subset of a diverse superfamily of enzymes built on a common protein kinase–like (PKL) fold. We explored this huge phylogenetic and functional space to cast light on the ancient evolution of this superfamily, its mechanistic core, and the structural basis for its observed diversity. We cataloged 27,677 ePKs and 18,699 ELKs, and classified them into 20 highly distinct families whose known members suggest regulatory functions. GOS data more than tripled the count of ELK sequences and enabled the discovery of novel families and classification and analysis of all ELKs. Comparison between and within families revealed ten key residues that are highly conserved across families. However, all but one of the ten residues has been eliminated in one family or another, indicating great functional plasticity. We show that loss of a catalytic lysine in two families is compensated by distinct mechanisms both involving other key motifs. This diverse superfamily serves as a model for further structural and functional analysis of enzyme evolution.

## Author Summary

The huge growth in sequence databases allows the characterization of every protein sequence by comparison with its relatives. Sequence comparisons can reveal both the key conserved functional motifs that define protein families and the variations specific to individual subfamilies, thus decorating any protein sequence with its evolutionary context. Inspired by the massive sequence trove from the Global Ocean Survey project, the authors looked in depth at the protein kinase–like (PKL) superfamily. Eukaryotic protein kinases (ePKs) are the pre-eminent controllers of eukaryotic cell biology and among the best studied of enzymes. By contrast, their prokaryotic relatives are much more poorly known. The authors hoped to both characterize and better understand these prokaryotic enzymes, and also, by contrast, provide insight into the core mechanisms of the eukaryotic protein kinases. The authors used remote homology methods, and bootstrapped on their discoveries to detect more than 45,000 PKL sequences. These clustered into 20 major families, of which the ePKs were just one. Ten residues are conserved between these families: 6 were known to be important in catalysis, but four more—including three highly conserved in ePKs—are still poorly understood, despite their ancient conservation. Extensive family-specific features were found, including the surprising loss of all but one of the ten key residues in one family or another. The authors explored some of these losses and found several cases in which changes in one key motif substitute for changes in another, demonstrating the plasticity of these sequences. Similar approaches can be used to better understand any other family of protein sequences.

## Abstract 3

Over 45,000 kinases, including 16,000 identified in the GOS expedition, were classified into 20 distinct families. This massive sequence comparison revealed a structural flexibility within eukaryotic protein kinases that helps explain their huge expansion in eukaryotes.

## Introduction

**Figure:**
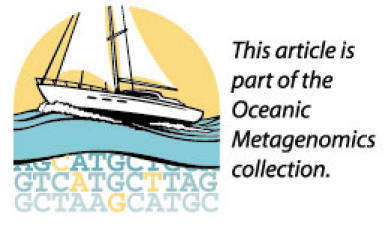


The eukaryotic protein kinase (ePK) domain is the most abundant catalytic domain in eukaryotic genomes and mediates the control of most cellular processes, by phosphorylation of a significant fraction of cellular proteins [1–3]. Most prokaryotic protein phosphorylation and signaling is thought to occur through structurally distinct histidine-aspartate kinases [4]. However, there is growing evidence for the existence and importance of different families of ePK-like kinases (ELKs) in prokaryotes [5–10]. ePKs and ELKs share the protein kinase–like (PKL) fold [11] and similar catalytic mechanisms, but ELKs generally display very low sequence identity (7%–17%) to ePKs and to each other. Crystal structures of ELKs such as aminoglycoside, choline, and Rio kinases reveal striking similarity to ePKs [12–14], and other ELKs have been defined by remote homology methods [6,15] and motif conservation [16]. Another set of even more divergent PKL kinases are undetectable by sequence methods, but retain structural and mechanistic conservation with ePKs. These include the phosphatidyl inositol kinases (PI3K) and related protein kinases, alpha kinases, the slime mold actin fragmin kinases, and the phosphatidyl inositol 5′ kinases [17–20].

These studies demonstrate that PKL kinases conserve both fold and catalytic mechanisms in the presence of tremendous sequence variation, which allows for an equivalent diversity in substrate binding and function. This makes the PKL fold a model system to investigate how sequence variation maps to functional specialization. Previous studies along these lines include the study of ePK-specific regulatory mechanisms, through ePK–ELK comparison [16], and the sequence determinants of functional specificity within one group (CMGC [CDK, MAPK, GSK3, and CLK kinases]) of ePKs [21].

Previous studies have been hampered by poor annotation and classification of ELK families and their low representation in sequence databases relative to ePKs. Recent large-scale microbial genomic sequencing, coupled with Global Ocean Sampling (GOS) metagenomic data, now allow a much more comprehensive analysis of these families. In particular, the GOS data provides more than 6 million new peptide sequences, mostly from marine bacteria [22,23], and more than triples the number of ELK sequences. Here, for the first time, we define the extent of 20 known and novel PKL families, define a set of ten key conserved residues within the catalytic domain, and explore specific elaborations that mediate the unique functions of distinct families. These highlight both underappreciated aspects of the catalytic core as well as unique family specific features, which in several cases reveal correlated changes that map to concerted variations in structure and mechanism.

## Results

### Discovery and Classification of PKL Kinase Families

**Figure 1:**
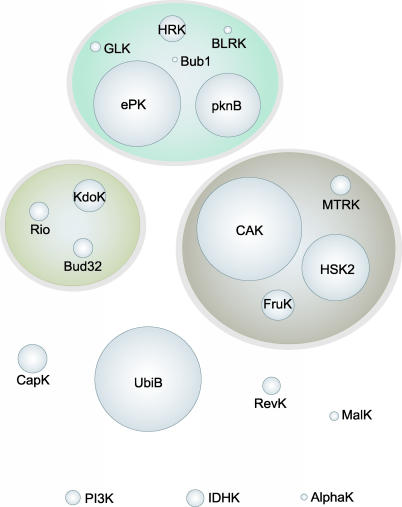
Sequence and Structure Based Clustering of PKL Families Despite minimal sequence similarity, relationships between families can be estimated by profile–profile matching and alignments restricted to conserved motifs. Three main clusters of families are seen (shaded ovals): CAK, ePK, and KdoK. Four more families (towards bottom) are distantly related to these clusters, while three more (PI3K, AlphaK, IDHK, at bottom) have no sequence similarity outside a subset of key motifs. The area of each sphere represents the family size within GOS data.

Kinase sequences were detected using hidden Markov model (HMM) profiles of known PKLs as well as with a motif model focused on key conserved PKL motifs [16,24]. Results of each approach were used to iteratively build, search, and refine new sets of HMMs, using both public and GOS data. Weak but significant sequence matches were used as seeds to define and elaborate novel families. The final result was 16,248 GOS sequences (Dataset S1) classified into 20 HMM-defined PKL families (Table 1; Figure 1; Dataset S2). A similar analysis of the National Center of Biotechnology Information nonredundant public database (NCBI-nr) revealed 24,924 ePK and 5,151 ELK sequences (Dataset S1). More than 1,400 of the NCBI ELK sequences were annotated as hypothetical or unknown, and several hundred more are misannotated or have no functional annotation. GOS data at least doubles the size of most families, and permits an in-depth analysis of family structure and conservation. Two families that are more than 10-fold enriched in GOS (CapK and HSK2) are found largely in α proteobacteria, which are also highly enriched in GOS. Both CapK and HRK contain viral-specific subfamilies that are also greatly GOS enriched, indicating that differences in kinase distribution between databases are largely due to taxonomic biases. As expected, eukaryotic-specific families, (ePK, Bub1, PI3K, AlphaK) are underrepresented in GOS.

**Table 1:**
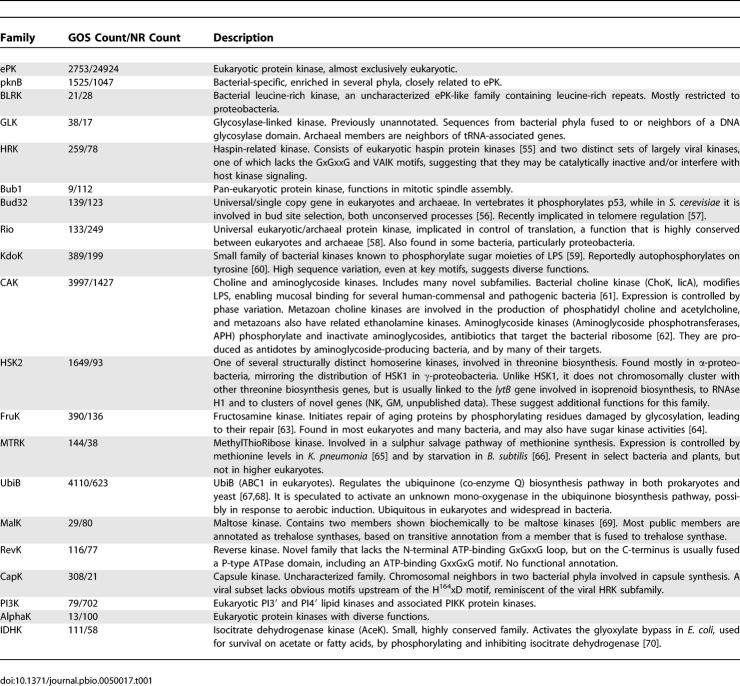
The 20 PKL Families, Their Gene Counts in GOS and NCBI-nr, and Functional Notes

### Functional Diversity of PKL Families

These 20 PKL families display great functional and sequence diversity, though common sequence motifs and functional themes recur. Some families are entirely uncharacterized, and few have been well studied, though most have some characterized members, many with known kinase activity. Their substrates include proteins and small molecules such as lipids, sugars, and amino acids, and they generally appear to have regulative functions (Table 1). This is in contrast to the diversity of several other structurally unrelated, small-molecule kinase families that play largely metabolic roles [25]. Profile–profile alignments show clear but distant relationships between several families, which are enclosed by ovals in Figure 1. The ePK cluster includes pknB, which is highly similar to but distinct from ePK and is distinguished by its exclusive bacterial specificity, as opposed to the mostly eukaryotic ePK family. The other major cluster is centered on the large and divergent CAK (choline and aminoglycoside kinase) family, and includes three other families of small-molecule kinases. CAK itself is particularly diverse, containing subfamilies that are specific for choline/ethanolamine and aminoglycosides, as well as many novel subfamilies, some of which are specific to eukaryotic sublineages. A looser cluster is formed between the Rio and Bud32 families, which are universal among both eukaryotes and archaeae, and the bacterial lipopolysaccharide kinase family KdoK. An additional four families (UbiB, revK, MalK, CapK) are distantly related to all three clusters, and are distinct from another set—PI3K, AlphaK, and IDHK—which have even less similarity to any other kinase; for PI3K and AlphaK, the relationship to kinases was determined by structural comparisons [11], while IDHK displays only conservation of the key residues and motifs found in all PKL kinases.

Sequence similarity between these 20 families varies from very low (~20%) to almost undetectable. Sequence-profile methods are generally required to align families within the oval clusters of Figure 1, while alignments between clusters require profile–profile methods. The diversity of this collection is demonstrated by comparison with the automated sequence- and profile-based clustering of the overall GOS analysis [22], which assigns 93% of these sequences into 32 clusters, each of which is largely specific to one of our 20 families.

### Key Conserved Residues Unify Diverse Kinase Families

**Figure 2:**
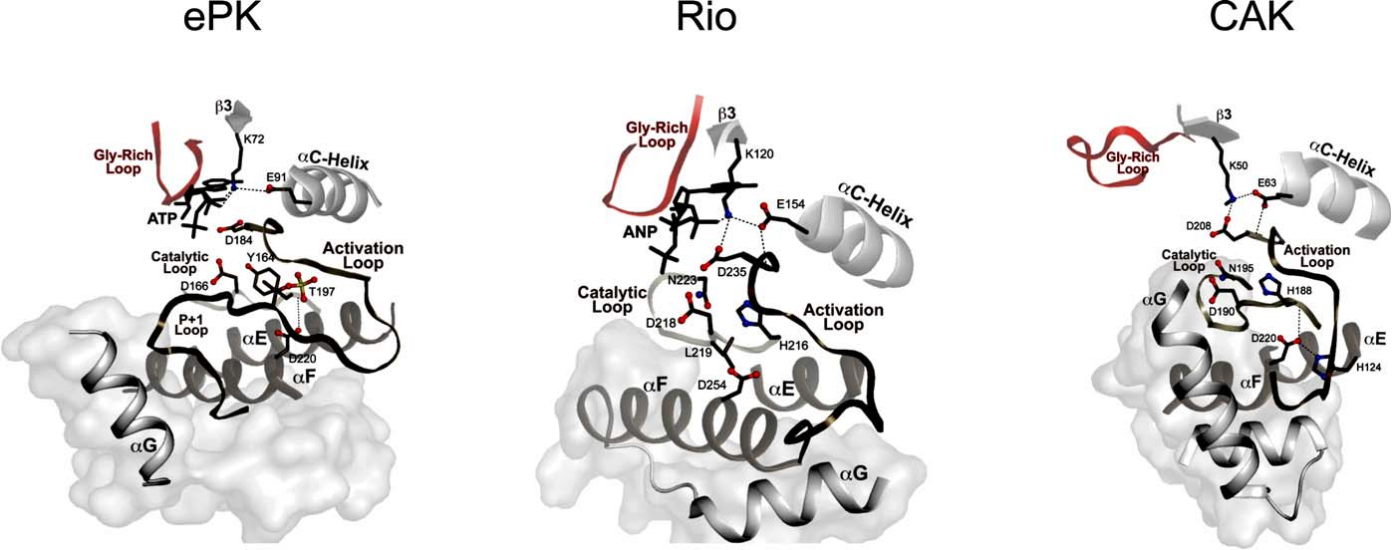
The Conserved Core and Variable Regions of the Catalytic Domain The conserved core in three distinct families, namely ePK (PKA [52]), Rio (A. fulgidis Rio2 [14]), and CAK (APH(3′)-IIIa [12]). The conserved regions are shown in ribbon representation and the variable regions in surface representation. The illustrations were created in PyMOL (http://www.pymol.org). Some highly conserved residues (see Figure 3) and their associated interactions are shown.

**Figure 3:**
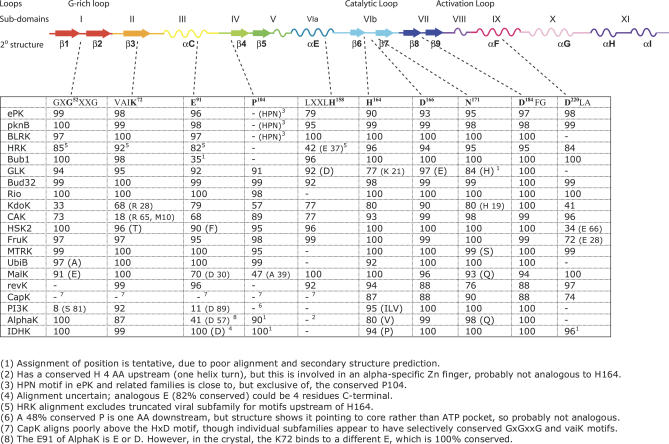
Conservation of Secondary Structure, Key Motifs, and Residues between Families The ePK secondary structure is shown with standard annotations of subdomains [53] and structural elements. Subdomains I–IX are generally conserved in all PKLs. Key residues are bolded and numbered; dashed lines point to positions within secondary structure elements. The table below shows the conservation (% identity) of the ten key residues, showing their broad conservation across families, but the successful replacement of almost all of them in at least one family. Parentheses indicate changes to another conserved residue and dashes indicate unconserved positions. Key residues are numbered based on their position in PKA: G52, K72, E91, P104 (V^PKA^), H158, H164 (Y^PKA^), D166, N171, D184, and D220. More detailed figures are shown in Dataset S3.

Comparison between all families reveals a set of ten key residues that not only account for one-third of the residues conserved within each family, but also are consistently conserved between families, constituting a core pattern of conservation that helps define this superfamily (Table 2, Figure 2, Figure 3). These residues are conserved across the major divisions of life, which diverged one to two billion years ago, and across diverse families, which presumably diverged even earlier. Thus, they are likely to mediate core functions of the catalytic domain rather than merely maintaining their structures. Six of these residues are known to be involved in ATP and substrate binding and catalysis (G52, K72, E91, D166, N171 D184; residues numbered based on PKA structure 1ATP except where otherwise noted; see Table 3). The full functions of the other four remain unclear, though three of them (H158, H164, and D220) are part of a hydrogen-bonding network that links the catalytically important DFG motif with substrate binding regions (Figure 2). The conservation of this network across diverse PKL structures suggested a role for this network in coupling DFG motif-associated conformational changes with substrate binding and release [16]. Despite this ancient conservation, different families of ePKs have lost individual members of this triad without destroying structure or catalytic function: H164 is changed to a tyrosine in PKA and many other AGC families; H158 is lost in most tyrosine kinases; and D220 is lost in the Pim family. The Pim1 structure retains an ePK-like structure, perhaps in part due to stabilization of the catalytic loop by the activation loop, a function normally performed by D220 [26], suggesting a novel mode of coupling ATP and substrate binding in this family. The individual loss of each member of this triad suggests that they have independent functions yet to be understood.

**Table 2:**
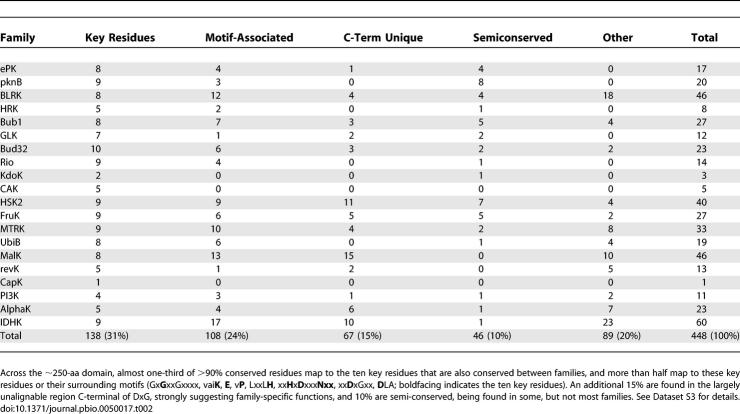
Distribution of Residues That Are >90% Identical within Each Family

**Table 3:**
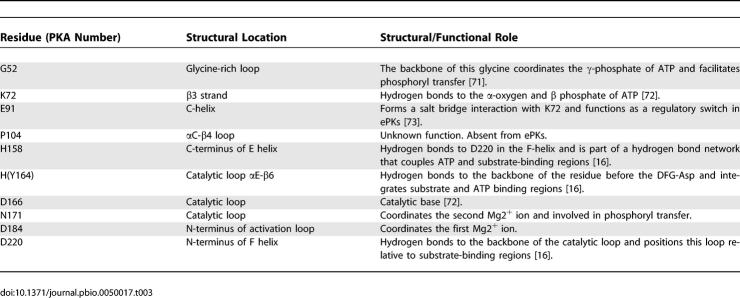
Structural/Functional Role of Highly Conserved Residues

### Sequence and Structural Diversity

**Figure 4:**
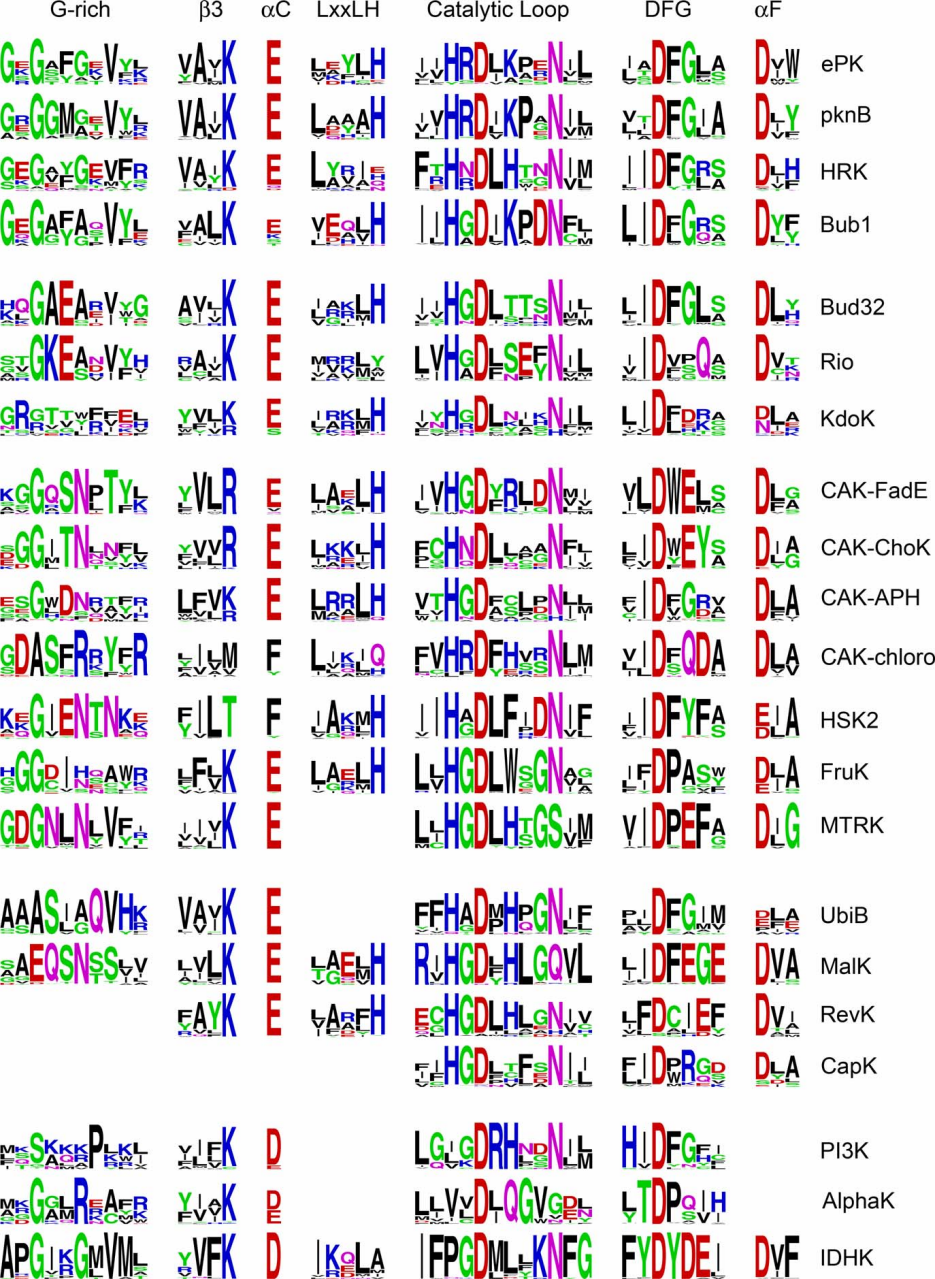
Sequence Logos Depicting Conservation of Core Motifs and Neighboring Sequences across Most Kinase Families and Selected CAK Subfamilies Motifs are GxGxxGxxxx, VAIK, E, LxxLH, xxHxDxxxxNxx, xxDFGxx, and Dxx. The size of the letters corresponds to their information content [54]. Families with less than 100 members (BLRK, GLK) are omitted. The diverse CAK family is represented by four distinct subfamilies: APH contains many aminoglycoside resistance kinases and ChoK includes most ChoKs, while FadE and chloro are less well described. For the HRK family, the first two motif logos omit the viral subfamily that lacks these motifs.

Family-specific functions are mediated by features that are highly conserved within families, but that are divergent between families (Figure 4). Many family-selective residues map to the motifs surrounding the ten key residues, or to the divergent C-terminal substrate-binding region (Tables 2 and S1). The proximity of these residues to the active site suggests that they are key in selecting substrates or tuning mechanism of action. For instance, the 4–amino acid (aa) stretch between the HxD^166^ and N^171^ residues is highly conserved but distinct between families (Figure 4), and provides a discriminative signature that defines each family. Within ePKs, tyrosine and serine/threonine-specific kinases display distinct patterns of conservation within this 4-aa stretch [27]. Serine/threonine kinases conserve a [LI]KPx motif within this stretch, while tyrosine kinases conserve a [LI]AAR motif. These variations alter the surface electrostatics of the substrate-binding pocket, thereby contributing to substrate specificity [27].

The C-terminal region of ~100 aa following the DFG motif is highly divergent between families, apart from the conserved D220 at the beginning of the F-helix (Figure 2; Dataset S3). Secondary structure is generally predicted to be helical, but the poor sequence conservation and known structures [11] suggest that the overall orientation of the helices may be different between families. Notably, in the crystal structures of APH bound to its substrate, kanamycin [28], the relative positioning of the substrate-binding helices (αH–αI) is distinct from that of ePKs (Figure 2). The presence of unique patterns of conservation in each family (Table 2) also suggests that this region is involved in family-specific functions.

Several families contain sizeable (~30–100 aa) insert segments between core subdomains that are specific to clusters of families. Most CAK members have an insert segment between subdomains VIa and VIb. There is very little sequence similarity within this segment across CAK members, but structures of APH and ChoK indicate some structural similarity and highlight its role in substrate binding [28,29]. An equivalent insert is seen in the other CAK cluster families, FruK, HSK2, and MTRK. Similarly, KdoK and Rio contain an insert between subdomains II and III, which shows some sequence similarity between these families. In the Rio2 structure, this insert is disordered, but the presence of a conserved threonine suggests a possible regulatory role [14]. This region also contains an insert in the distinct UbiB family. Finally, the ePK, pknB, and HRK families contain an extended activation loop between subdomains VIII and IX. These kinases are generally activated by phosphorylation of this loop, the negative charge of which helps to coordinate key structural elements during the activation process, including a family-selective HRD arginine in the catalytic loop [30,31].

### Mechanistic Diversity of the Catalytic Core

**Figure 5:**
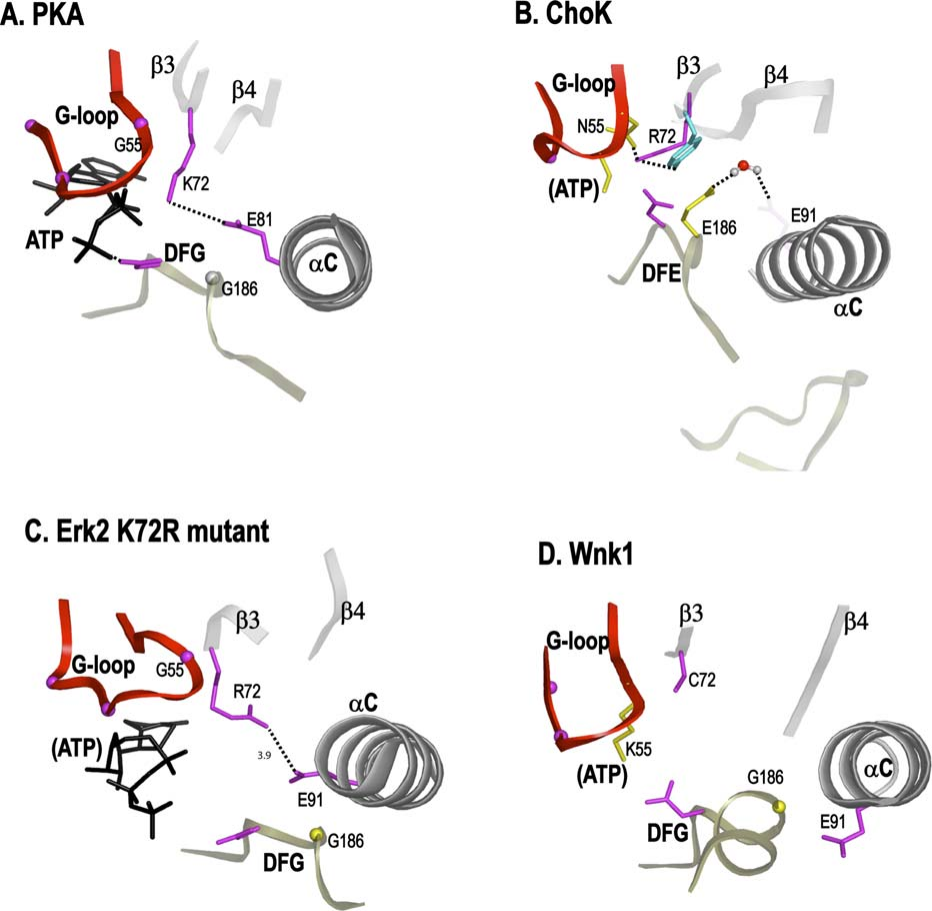
Mechanistic Diversity of the ATP-Binding Pocket. (A) PKA showing structural interactions associated with K72 in active ATP-bound state. The salt bridge interaction between K72 and E91 is shown by dotted lines. (B) Structural interactions associated with Arg111^ChoK^ in ChoK. (C) Conformational changes associated with Arg52^Erk2^ in the Erk2 mutant structure. Here, the arginine does not form a salt bridge interaction with Glu69^Erk2^ (E91), but moves closer towards Glu69^Erk2^ upon ATP binding. (D) Inactive state of Wnk1: K72 is shifted over to the G-loop (K233^Wnk1^) and E91 (Glu268^Wnk1^) hydrogen bonds to a conserved Arg (R348^Wnk1^ within the HRD motif) in the catalytic loop. (A–D) Residues conserved across all the major families are colored in magenta, while family-specific residues are colored in gold. Hydrogen bonds are indicated in dotted lines.

A surprising finding was that while ten key residues are conserved both within and between families, all but one of them was dispensable in one family or another (Figure 3), indicating that even catalytic residues are malleable in the appropriate context. Here we explore the effect of loss of the “catalytic lysine” K72, which typically positions the α and β phosphates of ATP (Figure 5A). Mutation of this lysine in ePKs is a common method to make inactive kinases [32]. Yet this residue is conserved as an arginine (R111^ChoK^) in most CAK subfamilies, as a methionine in the CAK-chloro subfamily, and as a threonine in the related HSK2 family (Figure 4).

In the two major CAK subfamilies with a conserved R72 (FadE and choline kinase [ChoK]), we see correlated changes in the glycine-rich and DFG motifs (Figure 4). Specifically, the Phe and Gly within the GxGxFG motif (F54 and G55) are changed to Ser/Thr and Asn, respectively (S86^ChoK^, N87^ChoK^), and G186 within the DFG motif is changed to E. Both the GxGxFG and DFG motifs are spatially proximal to K72 (Figure 5A). Thus, correlated changes in these two motifs could structurally account for the K-to-R change. Indeed, in the ChoK crystal structure [13], N55 protrudes into the ATP binding pocket, and hydrogen bonds to R72. In addition, the conserved E91 in helix C, which typically forms a salt bridge with K72, is hydrogen bonded (via a water molecule) to the covarying E186, thus linking these three correlated changes and stabilizing R72 in a unique conformation (Figure 5B). By contrast, the two solved APH structures (1ND4 and 2BKK) retain the “ancestral” sequence state with K72 and G186, and lack N55.

Mutation of R72 or E186 to alanine in ChoK reduces the catalytic rate by several fold [33]. To test the possible role of these residues in the ChoK catalytic mechanism, we modeled an ATP in the active site of ChoK (based on the nucleotide-bound structures of APH and PKA). This revealed that R72 partially occludes the ATP binding site and is likely to move upon ATP binding. Notably, a K72-to-R mutation in Erk2 [34] also exhibits a conformational change in R72 upon nucleotide binding (Figure 5C). A similar conformational change in ChoK upon ATP binding could result in formation of a R72–E91 salt bridge similar to the activation of ePKs (Figure 5A). In this conformation, R72 could potentially hydrogen bond to both E91 as well as to the covarying E186 in ChoKs, which might explain the covariation of R72 and E186 in these families.

### Variation on a Theme

Other CAK members display distinct coordinated changes at the G55, K72, and G186 positions. The chloro subfamily of CAK loses the positive charge at position 72 altogether, replacing it with methionine, and has concurrent changes to R55 and Q186 (Figure 4). This may reflect a shift of the positive charge from position 72 to 55, an event that also happened in Wnk kinases, the only functional ePK family that lacks K72. The conserved K55 of Wnks is required for catalysis and has been shown to interact with ATP similarly to K72 of PKA [35] (Figure 5D). Hence, two evolutionary inventions may have converted the same core motif residue from one function to another. In CAK-chloro, the unpaired E91 position loses its charge to become a conserved Phe. The function of this Phe is unknown, but is likely to be important since it is also conserved in HSK2, a related family, and the only other kinase family to conserve a Phe at the E91 position (Figure 4).

### Evolution of Conformational Flexibility and Regulation in ePKs

**Figure 6:**
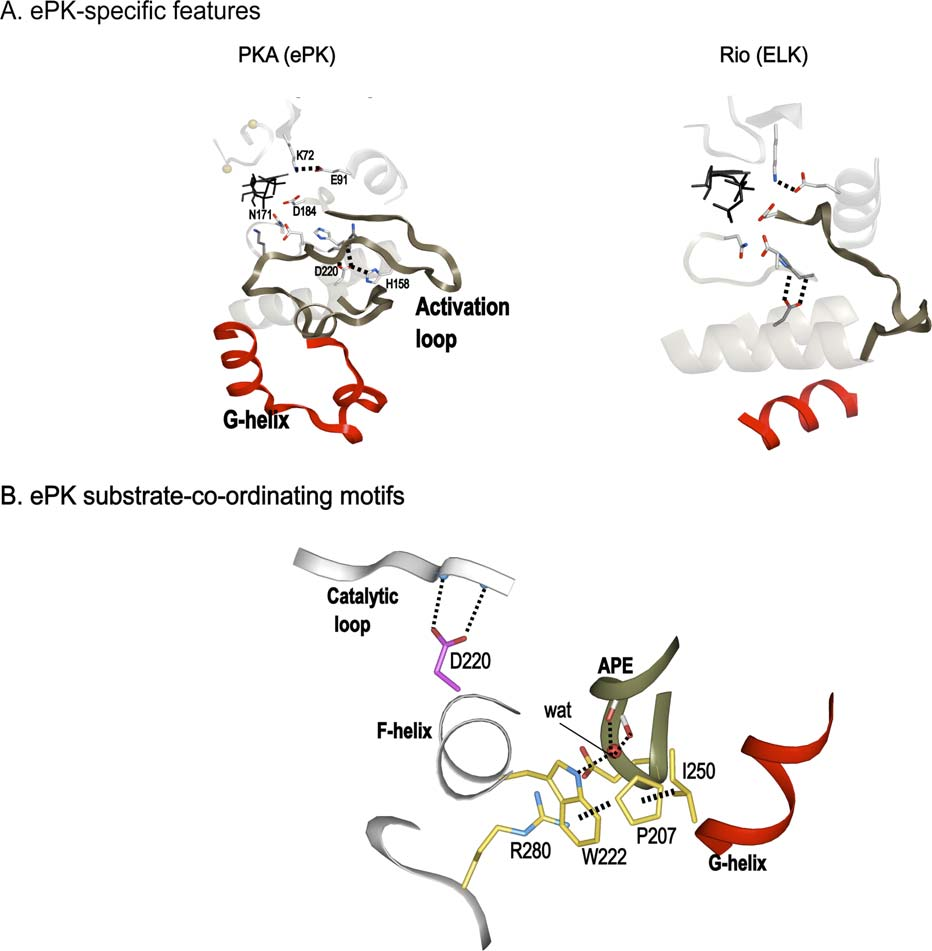
ePK-Specific Motifs and Interactions in the Substrate-Binding Region (A) The ePK-specific activation loop and G-helix are shown in PKA (PKA [52]). The corresponding regions are shown in Rio (A. fulgidis Rio2 [14]). The activation loop and G-helix are colored in red, and the core-conserved residues are shown in stick representation. (B) The three ePK-specific motifs in the C-terminal substrate-binding lobe and their structural interactions are shown. Hydrogen bonds are indicated by dotted lines. The conserved buried water is shown in CPK representation.

The ePK catalytic domain is highly flexible and undergoes extensive conformational changes upon ATP binding [36]. In contrast, crystal structures of APH, solved in both ATP-bound and -unbound forms, revealed modest structural changes in the ATP-binding pocket [37]. This difference in conformational flexibility is reflected in the patterns of conservation at key positions within the ATP-binding glycine-rich loop (Figure 4). Specifically, two conserved glycines (G50 and G55), which contribute to the conformational flexibility of this loop in ePKs, are replaced by non-glycines in APH. These two glycines are absent in several PKL families (Figure 4) while G52, which is involved in catalysis, is present in most, suggesting that the conformational flexibility of the nucleotide-binding loop is a feature of selected PKL families such as ePKs. Since conformational flexibility allows for regulation, it is likely that modest structural changes associated with nucleotide binding gradually evolved into quite dramatic structural rearrangements required to ensure that key players in various signaling pathways act only at the right place and at the right time. The conserved glycine (G186) within the catalytically important DFG motif may likewise have evolved for regulatory functions in ePKs [38]. This glycine is highly conserved in the ePK cluster but is absent from most other PKL families. However, within the small subfamily of magnesium-dependent Mnk ePK kinases, G185 is changed to aspartate (DFD). In the Mnk2 crystal structure, this DFD motif adopts an “out” conformation in which F185 protrudes into the ATP-binding site. This is in contrast to the “in” conformation, where it packs up below the C-helix [39]. Mutation of the Mnk2 D186 “back” to glycine results in both in and out conformations of the DFG motif, supporting the role of G186 in DFG-associated conformational changes. Such conformational transitions may facilitate regulation of activity since the conformation of the catalytic aspartate is also changed during this transition [38]. This may also explain why the ePK-specific extended activation loop, which is phosphorylated and undergoes dramatic conformational changes, is directly attached to the DFG motif (Figure 6A).

In addition to the flexible catalytic core, the substrate-binding regions appear to have evolved for tight regulation of ePK activity. In particular, the conserved G helix, which was recently shown to undergo a conformational changes upon substrate binding [40], is uniquely oriented in ePK/pknB (Figure 6A). Several ePK-conserved residues and motifs are at the interface between the G helix and the catalytic core (Figure 6B). These include the APE motif, located at the C-terminal end of the activation loop, a W-[SA]-X-[G] motif in the F-helix, and an arginine (R280), at the beginning of the I helix (Figure 6B). These three motifs structurally interact with each other and form a network that couples the substrate- and ATP-binding regions (Figure 6B). This network also involves conserved buried water molecules, which are known to contribute to the conformational flexibility of proteins [41]. Thus, this ePK/pknB-conserved network may also facilitate regulation by increasing the conformational flexibility of the substrate-binding regions [16].

## Discussion

Data from the GOS voyage provides a huge increase in available sequences for most prokaryotic gene families, enabling new studies in discovery, classification, and evolutionary and structural analysis of a wide array of gene families. Even for a eukaryotic family such as ePK kinases, GOS provides insights by greatly increasing understanding of related PKL families. GOS increases the number of known ELK sequences more than 3-fold, and has enabled both the discovery of novel families of kinases as well as a detailed analysis of conservation patterns and subfamilies within known families. We believe that the GOS data, coupled with the recent strong growth in whole-genome sequencing, provide the opportunity for similar insights into virtually every gene family with prokaryotic relatives.

PKL kinases are largely involved in regulatory functions, as opposed to the metabolic activities of other kinases with different folds [25]. The characteristics of this fold that lead to the explosion of diverse regulatory functions of eukaryotic ePKs have also been exploited for many different functions within prokaryotes. While these kinases reflect only ~0.25% of genes in both GOS and microbial genomes (ePKs represent ~2% of eukaryotic genes [42]), indicating a simpler prokaryotic lifestyle, they now outnumber the count of ~12,000 histidine kinases that we observe in GOS [22], suggesting that ELKs may be at least as important in bacterial cellular regulation as the “canonical” histidine kinases.

PKL kinases cross huge phylogenetic and functional spaces while still retaining a common fold and biochemical function of ATP-dependent phosphorylation. The presence of Rio and Bud32 genes in all eukaryotic and archaeal genomes suggests that at least this cluster dates back to the common ancestor of these domains of life. Similarly, the presence of UbiB in all eukaryotes and most bacterial groups, the close similarity of pknB/ePK families, and the widespread bacterial/eukaryotic distribution of FruK suggest their origins before the emergence of eukaryotes, or from an early horizontal transfer. Their ancient divergence leaves little or no trace of their shared structure within their protein sequence other than at functional motifs, which include a set of ten key residues that are highly conserved across all PKLs.

Despite the huge attention paid to ePKs, four key residues (P^104^, H^158^, H^164^, D^220^), three of which are highly conserved in ePKs, are still functionally obscure and worthy of greater attention, both in ELKs and ePKs. Conversely, it appears that nine of the ten key residues have been eliminated or transformed in individual families while maintaining fold and function, showing that almost anything is malleable in evolution given the right context. That right context is frequently a set of additional changes in the family-specific motifs surrounding these key residues, and we see that in the case of K72, a substitution to arginine triggers a cascade of other core substitutions that serve to retain basic function, while a substitution to methionine involves a shift of the positive charge normally provided by K72 to another conserved residue, in both CAK-chloro and Wnk kinases. Other core changes are also seen independently in very distinct families, such as the G55-to-A change in UbiB and the chloro subfamily of CAK, or the E91-to-F change in both chloro and HSK2, suggesting that these kinases are sampling a limited space of functional replacements.

These families vary greatly in diversity. While the ePK family has expanded to scores of deeply conserved functions [42], other families, including Bud32, Rio, Bub1, and UbiB, usually have just one or a handful of members per genome, suggesting critical function but an inability to innovate. The largely prokaryotic CAK family is also functionally and structurally diverse, containing several known functions and many distinct subfamilies likely to have novel functions. The diversity of both CAK and KdoK sequences may be related to their involvement in antibiotic resistance and immune evasion, likely to be evolutionarily accelerated processes. Comparison of CAK to the related and more functionally constrained HSK2, FruK, and MTRK families may reveal adaptive changes such as the ePK-specific flexibility changes that may assist in its diversity of functions.

GOS data are rich in highly divergent viral sequences, and accordingly we find a number of new subfamilies of viral kinases, including two of the three subfamilies of HRK and a subfamily of CapK. In both cases we see loss of N-terminal–conserved elements, suggesting that these kinases may have alternative functions or even act as inactive competitors to host kinases.

These patterns of sequence conservation and diversity raise many questions that can only be fully addressed by structural methods. The combination of structural and phylogenetic insights for ChoK enabled insights that were not clear from the structure alone, and enabled us to reject other inferences from the crystal structure that were not conserved within this family, highlighting the value of combining these approaches. The relative ease of crystallization of PKL domains, the emergence of high-throughput structural genomics, and our understanding of the diversity of these families make them attractive targets for structure determination of selected members, and position this family as a model for analysis of deep structural and functional evolution.

## Materials and Methods

### Discovery and classification of kinase genes.

Sequences used consisted of 17,422,766 open reading frames from GOS, 3,049,695 predicted open reading frames from prokaryotic genomes, and 2,317,995 protein sequences from NCBI-nr of February 10, 2005, as described [22]. Profile HMM searches were performed with a Time Logic Decypher system (Active Motif, http://timelogic.com) using in-house profiles for ePK, Haspin, Bub1, Bud32, Rio, ABC1 (UbiB), PI3K, and AlphaK domains, as well as Pfam profiles [43] for ChoK, APH, KdoK, and FruK, and TIGRFAM profiles [44] for HSK2 (thrB_alt), UbiB, and MTRK. A number (69) of additional ePK-annotated models from Superfamily 1.67 [45] were used to capture initial hits but not for further classification. Initial hits were clustered and re-run against all models, and each model was rebuilt and rerun three to seven times using ClustalW [46], MUSCLE [47], and hmmalign (http://hmmer.janelia.org) to align, followed by manual adjustment of alignments using Clustal and Pfaat [48] and model building with hmmbuild. Low-scoring members of each family (*e* > 1 × 10^−5^) were used as seeds to build new putative families, and profile–profile and sequence–profile alignments were used to merge families into a minimal set (Dataset S2). A motif-based Markov chain Monte Carlo multiple alignment model [49] based on the conserved motifs of Figure 3 was run independently and used to verify HMM hits and seed new potential families for blast-based clustering, model building, and examination for conserved residues. Final family assignment was by scoring against the set of HMM models, with manual examination of sequences with borderline scores (*e* > 1 × 10^−5^ or difference in *e-*values between best two models >.01).

### Family annotations.

Annotations of chromosomal neighbors used SMART [50] and a custom analysis of GOS neighbors ([22]; C. Miller, H. Li, D. Eisenberg, unpublished data). Annotation analysis was based on GenBank annotations and PubMed references. Taxonomic analysis used a mapping of GOS scaffolds to taxonomic groupings [22] and NCBI taxonomy tools.

### Family alignments and logos.

Residue conservation (Dataset S3) was counted from the final alignment using a custom script that omitted gap counts. These counts were then used to construct family logos using WebLogo (http://weblogo.berkeley.edu; [51]).

### Family comparisons.

Relatedness between families was estimated using several methods. HMM–HMM alignments and scores were computed using PRC (http://supfam.org/PRC), and sequence–profile alignments using hmmalign were analyzed using custom scripts and by inspection. Both full-length and motif multiple alignments were also created and used for the family comparisons.

## Supporting Information

Dataset S1 FastA-Formatted Sequence Files for Each of the 20 Kinase Families, Including Both GOS and Public Sequences (10 MB BZ2)

Dataset S2 HMM Profiles for the 20 Kinase Families in HMMer Format (2.4 MB HMM)

Dataset S3 Domain Profiles for 20 PKL Families These 20 spreadsheets show the conservation profile at each residue of the kinase domain for each family, including annotations and classifications of individual residues. Each worksheet details the alignment of one kinase family to its HMM. Every row corresponds to a position within the alignment, listing the four most common amino acids (aa) in that row along with their fractional popularity. The number of aa's and number of gaps at that position within the alignment is also listed. The “Notes” column annotates conservation status of selected residues and other notes, while the “>90% Conserved” annotates those corresponding residues as to their class (Core, Motif, Motif-Associated, Semi-Conserved, C-terminal, Unique, or external to the kinase domain). A number of color highlights are used. (1) Positions with few aa's in the alignment (typically inserts within the domain that are not of great interest) are shaded gray: typically dark gray for ≤20 aa at that position, and light gray for >20 but still low (the range varies depending on the depth of the alignment). Rows highlighted in gray have no highlights in any other columns and are assumed not to be part of the core domain. (2) Core motifs are highlighted in bold and blue. (3) The fractional count for the most popular aa is labeled green if 1, dark yellow if >0.9, and light yellow if >0.8 and <0.9.
(1.6 MB XLS)

### Accession Numbers

The Protein Databank (http://www.pdb.org) accession numbers for the structures discussed in this paper are PKA (1ATP), A. fulgidis Rio2 (1TQP), C. elegans choline kinase (INW1), Erk2 (1GOL), Wnk1 (1T4H), and APH(3′)-IIIa (1J7L). The Pfam (http://pfam.cgb.ki.se) accession numbers for the structures discussed in this paper are ChoK (PF01633.8), APH (PF01636.9), KdoK (PF06293.3), and FruK (PF03881.4). The TIGRFAM (http://www.tigr.org/TIGRFAMs) accession numbers for the structures discussed in this paper are HSK2 (TIGR00938), UbiB (TIGR01982), and MTRK (TIGR01767).

## Abbreviations

aa: amino acid
CAK: choline and aminoglycoside kinase
ChoK: choline kinase
ELK: ePK-like kinase
ePK: eukaryotic protein kinase
GOS: Global Ocean Sampling
HMM: hidden Markov model
NCBI: National Center of Biotechnology Information
PKL: protein kinase–like

## References

[pbio-0050017-b001] Manning G, Whyte DB, Martinez R, Hunter T, Sudarsanam S (2002). The protein kinase complement of the human genome. Science.

[pbio-0050017-b002] Cohen P (2002). Protein kinases—The major drug targets of the twenty-first century?. Nat Rev Drug Discov.

[pbio-0050017-b003] Manning G, Plowman GD, Hunter T, Sudarsanam S (2002). Evolution of protein kinase signaling from yeast to man. Trends Biochem Sci.

[pbio-0050017-b004] Parkinson JS (1993). Signal transduction schemes of bacteria. Cell.

[pbio-0050017-b005] Kennelly PJ, Potts M (1999). Life among the primitives: Protein O-phosphatases in prokaryotes. Front Biosci.

[pbio-0050017-b006] Leonard CJ, Aravind L, Koonin EV (1998). Novel families of putative protein kinases in bacteria and archaea: Evolution of the “eukaryotic” protein kinase superfamily. Genome Res.

[pbio-0050017-b007] Krupa A, Srinivasan N (2005). Diversity in domain architectures of Ser/Thr kinases and their homologues in prokaryotes. BMC Genomics.

[pbio-0050017-b008] Zhang CC (1996). Bacterial signalling involving eukaryotic-type protein kinases. Mol Microbiol.

[pbio-0050017-b009] Young TA, Delagoutte B, Endrizzi JA, Falick AM, Alber T (2003). Structure of Mycobacterium tuberculosis PknB supports a universal activation mechanism for Ser/Thr protein kinases. Nat Struct Biol.

[pbio-0050017-b010] Kennelly PJ (2002). Protein kinases and protein phosphatases in prokaryotes: A genomic perspective. FEMS Microbiol Lett.

[pbio-0050017-b011] Scheeff ED, Bourne PE (2005). Structural evolution of the protein kinase-like superfamily. PLoS Comput Biol.

[pbio-0050017-b012] Hon WC, McKay GA, Thompson PR, Sweet RM, Yang DS (1997). Structure of an enzyme required for aminoglycoside antibiotic resistance reveals homology to eukaryotic protein kinases. Cell.

[pbio-0050017-b013] Peisach D, Gee P, Kent C, Xu Z (2003). The crystal structure of choline kinase reveals a eukaryotic protein kinase fold. Structure (Camb).

[pbio-0050017-b014] LaRonde-LeBlanc N, Wlodawer A (2004). Crystal structure of A. fulgidus Rio2 defines a new family of serine protein kinases. Structure.

[pbio-0050017-b015] Cheek S, Ginalski K, Zhang H, Grishin NV (2005). A comprehensive update of the sequence and structure classification of kinases. BMC Struct Biol.

[pbio-0050017-b016] Kannan N, Neuwald AF (2005). Did protein kinase regulatory mechanisms evolve through elaboration of a simple structural component?. J Mol Biol.

[pbio-0050017-b017] Grishin NV (1999). Phosphatidylinositol phosphate kinase: A link between protein kinase and glutathione synthase folds. J Mol Biol.

[pbio-0050017-b018] Walker EH, Pacold ME, Perisic O, Stephens L, Hawkins PT (2000). Structural determinants of phosphoinositide 3-kinase inhibition by wortmannin, LY294002, quercetin, myricetin, and staurosporine. Mol Cell.

[pbio-0050017-b019] Steinbacher S, Hof P, Eichinger L, Schleicher M, Gettemans J (1999). The crystal structure of the Physarum polycephalum actin-fragmin kinase: An atypical protein kinase with a specialized substrate-binding domain. EMBO J.

[pbio-0050017-b020] Yamaguchi H, Matsushita M, Nairn AC, Kuriyan J (2001). Crystal structure of the atypical protein kinase domain of a TRP channel with phosphotransferase activity. Mol Cell.

[pbio-0050017-b021] Kannan N, Neuwald AF (2004). Evolutionary constraints associated with functional specificity of the CMGC protein kinases MAPK, CDK, GSK, SRPK, DYRK, and CK2{alpha}. Protein Sci.

[pbio-0050017-b022] Yooseph S, Sutton G, Rusch DB, Halpern AL, Williamson SJ (2007). The *Sorcerer II* Global Ocean Sampling expedition: Expanding the universe of protein families. PLoS Biol.

[pbio-0050017-b023] Rusch DB, Halpern AL, Sutton G, Heidelberg KB, Williamson S (2007). The *Sorcerer II* Gobal Ocean Sampling expedition: Northwest Atlantic through eastern tropical Pacific. PLoS Biol.

[pbio-0050017-b024] Neuwald AF, Liu JS, Lawrence CE (1995). Gibbs motif sampling: Detection of bacterial outer membrane protein repeats. Protein Sci.

[pbio-0050017-b025] Cheek S, Zhang H, Grishin NV (2002). Sequence and structure classification of kinases. J Mol Biol.

[pbio-0050017-b026] Qian KC, Wang L, Hickey ER, Studts J, Barringer K (2005). Structural basis of constitutive activity and a unique nucleotide binding mode of human Pim-1 kinase. J Biol Chem.

[pbio-0050017-b027] Taylor SS, Radzio-Andzelm E, Hunter T (1995). How do protein kinases discriminate between serine/threonine and tyrosine? Structural insights from the insulin receptor protein-tyrosine kinase. FASEB J.

[pbio-0050017-b028] Nurizzo D, Shewry SC, Perlin MH, Brown SA, Dholakia JN (2003). The crystal structure of aminoglycoside-3′-phosphotransferase-IIa, an enzyme responsible for antibiotic resistance. J Mol Biol.

[pbio-0050017-b029] Thompson PR, Schwartzenhauer J, Hughes DW, Berghuis AM, Wright GD (1999). The COOH terminus of aminoglycoside phosphotransferase (3′)-IIIa is critical for antibiotic recognition and resistance. J Biol Chem.

[pbio-0050017-b030] Nolen B, Taylor S, Ghosh G (2004). Regulation of protein kinases: Controlling activity through activation segment conformation. Mol Cell.

[pbio-0050017-b031] Boitel B, Ortiz-Lombardia M, Duran R, Pompeo F, Cole ST (2003). PknB kinase activity is regulated by phosphorylation in two Thr residues and dephosphorylation by PstP, the cognate phospho-Ser/Thr phosphatase, in Mycobacterium tuberculosis. Mol Microbiol.

[pbio-0050017-b032] Gibbs CS, Zoller MJ (1991). Rational scanning mutagenesis of a protein kinase identifies functional regions involved in catalysis and substrate interactions. J Biol Chem.

[pbio-0050017-b033] Yuan C, Kent C (2004). Identification of critical residues of choline kinase A2 from Caenorhabditis elegans. J Biol Chem.

[pbio-0050017-b034] Robinson MJ, Harkins PC, Zhang J, Baer R, Haycock JW (1996). Mutation of position 52 in ERK2 creates a nonproductive binding mode for adenosine 5′-triphosphate. Biochemistry.

[pbio-0050017-b035] Xu B, English JM, Wilsbacher JL, Stippec S, Goldsmith EJ (2000). WNK1, a novel mammalian serine/threonine protein kinase lacking the catalytic lysine in subdomain II. J Biol Chem.

[pbio-0050017-b036] Akamine P, Madhusudan, Wu J, Xuong NH, Ten Eyck LF (2003). Dynamic features of cAMP-dependent protein kinase revealed by apoenzyme crystal structure. J Mol Biol.

[pbio-0050017-b037] Thompson PR, Boehr DD, Berghuis AM, Wright GD (2002). Mechanism of aminoglycoside antibiotic kinase APH(3′)-IIIa: Role of the nucleotide positioning loop. Biochemistry.

[pbio-0050017-b038] Levinson NM, Kuchment O, Shen K, Young MA, Koldobskiy M (2006). A SRC-like inactive conformation in the abl tyrosine kinase domain. PLoS Biol.

[pbio-0050017-b039] Jauch R, Jakel S, Netter C, Schreiter K, Aicher B (2005). Crystal structures of the Mnk2 kinase domain reveal an inhibitory conformation and a zinc binding site. Structure.

[pbio-0050017-b040] Dar AC, Dever TE, Sicheri F (2005). Higher-order substrate recognition of eIF2alpha by the RNA-dependent protein kinase PKR. Cell.

[pbio-0050017-b041] Fischer S, Verma CS (1999). Binding of buried structural water increases the flexibility of proteins. Proc Natl Acad Sci U S A.

[pbio-0050017-b042] Goldberg JM, Manning G, Liu A, Fey P, Pilcher KE (2006). The dictyostelium kinome—Analysis of the protein kinases from a simple model organism. PLoS Genet.

[pbio-0050017-b043] Bateman A, Birney E, Cerruti L, Durbin R, Etwiller L (2002). The Pfam protein families database. Nucleic Acids Res.

[pbio-0050017-b044] Haft DH, Loftus BJ, Richardson DL, Yang F, Eisen JA (2001). TIGRFAMs: A protein family resource for the functional identification of proteins. Nucleic Acids Res.

[pbio-0050017-b045] Gough J, Karplus K, Hughey R, Chothia C (2001). Assignment of homology to genome sequences using a library of hidden Markov models that represent all proteins of known structure. J Mol Biol.

[pbio-0050017-b046] Thompson JD, Higgins DG, Gibson TJ (1994). CLUSTAL W: Improving the sensitivity of progressive multiple sequence alignment through sequence weighting, position-specific gap penalties and weight matrix choice. Nucleic Acids Res.

[pbio-0050017-b047] Edgar RC (2004). MUSCLE: Multiple sequence alignment with high accuracy and high throughput. Nucleic Acids Res.

[pbio-0050017-b048] Johnson JM, Mason K, Moallemi C, Xi H, Somaroo S (2003). Protein family annotation in a multiple alignment viewer. Bioinformatics.

[pbio-0050017-b049] Neuwald AF, Liu JS (2004). Gapped alignment of protein sequence motifs through Monte Carlo optimization of a hidden Markov model. BMC Bioinformatics.

[pbio-0050017-b050] Schultz J, Copley RR, Doerks T, Ponting CP, Bork P (2000). SMART: A web-based tool for the study of genetically mobile domains. Nucleic Acids Res.

[pbio-0050017-b051] Crooks GE, Hon G, Chandonia JM, Brenner SE (2004). WebLogo: A sequence logo generator. Genome Res.

[pbio-0050017-b052] Knighton DR, Zheng JH, Ten Eyck LF, Ashford VA, Xuong NH (1991). Crystal structure of the catalytic subunit of cyclic adenosine monophosphate-dependent protein kinase. Science.

[pbio-0050017-b053] Hanks SK, Quinn AM, Hunter T (1988). The protein kinase family: Conserved features and deduced phylogeny of the catalytic domains. Science.

[pbio-0050017-b054] Neuwald AF, Kannan N, Poleksic A, Hata N, Liu JS (2003). Ran's C-terminal, basic patch, and nucleotide exchange mechanisms in light of a canonical structure for Rab, Rho, Ras, and Ran GTPases. Genome Res.

[pbio-0050017-b055] Higgins JM (2001). Haspin-like proteins: A new family of evolutionarily conserved putative eukaryotic protein kinases. Protein Sci.

[pbio-0050017-b056] Facchin S, Lopreiato R, Ruzzene M, Marin O, Sartori G (2003). Functional homology between yeast piD261/Bud32 and human PRPK: Both phosphorylate p53 and PRPK partially complements piD261/Bud32 deficiency. FEBS Lett.

[pbio-0050017-b057] Downey M, Houlsworth R, Maringele L, Rollie A, Brehme M (2006). A genome-wide screen identifies the evolutionarily conserved KEOPS complex as a telomere regulator. Cell.

[pbio-0050017-b058] LaRonde-LeBlanc N, Wlodawer A (2005). The RIO kinases: An atypical protein kinase family required for ribosome biogenesis and cell cycle progression. Biochim Biophys Acta.

[pbio-0050017-b059] White KA, Lin S, Cotter RJ, Raetz CR (1999). A Haemophilus influenzae gene that encodes a membrane bound 3-deoxy-D-manno-octulosonic acid (Kdo) kinase. Possible involvement of kdo phosphorylation in bacterial virulence. J Biol Chem.

[pbio-0050017-b060] Zhao X, Lam JS (2002). WaaP of Pseudomonas aeruginosa is a novel eukaryotic type protein-tyrosine kinase as well as a sugar kinase essential for the biosynthesis of core lipopolysaccharide. J Biol Chem.

[pbio-0050017-b061] Serino L, Virji M (2000). Phosphorylcholine decoration of lipopolysaccharide differentiates commensal *Neisseriae* from pathogenic strains: Identification of licA-type genes in commensal *Neisseriae*. Mol Microbiol.

[pbio-0050017-b062] Wright GD, Thompson PR (1999). Aminoglycoside phosphotransferases: Proteins, structure, and mechanism. Front Biosci.

[pbio-0050017-b063] Delpierre G, Collard F, Fortpied J, Van Schaftingen E (2002). Fructosamine 3-kinase is involved in an intracellular deglycation pathway in human erythrocytes. Biochem J.

[pbio-0050017-b064] Fortpied J, Gemayel R, Stroobant V, van Schaftingen E (2005). Plant ribulosamine/erythrulosamine 3-kinase, a putative protein-repair enzyme. Biochem J.

[pbio-0050017-b065] Tower PA, Alexander DB, Johnson LL, Riscoe MK (1993). Regulation of methylthioribose kinase by methionine in Klebsiella pneumoniae. J Gen Microbiol.

[pbio-0050017-b066] Sekowska A, Mulard L, Krogh S, Tse JK, Danchin A (2001). MtnK, methylthioribose kinase, is a starvation-induced protein in Bacillus subtilis. BMC Microbiol.

[pbio-0050017-b067] Poon WW, Davis DE, Ha HT, Jonassen T, Rather PN (2000). Identification of Escherichia coli ubiB, a gene required for the first monooxygenase step in ubiquinone biosynthesis. J Bacteriol.

[pbio-0050017-b068] Do TQ, Hsu AY, Jonassen T, Lee PT, Clarke CF (2001). A defect in coenzyme Q biosynthesis is responsible for the respiratory deficiency in Saccharomyces cerevisiae abc1 mutants. J Biol Chem.

[pbio-0050017-b069] Jarling M, Cauvet T, Grundmeier M, Kuhnert K, Pape H (2004). Isolation of mak1 from Actinoplanes missouriensis and evidence that Pep2 from Streptomyces coelicolor is a maltokinase. J Basic Microbiol.

[pbio-0050017-b070] Cozzone AJ, El-Mansi M (2005). Control of isocitrate dehydrogenase catalytic activity by protein phosphorylation in Escherichia coli. J Mol Microbiol Biotechnol.

[pbio-0050017-b071] Aimes RT, Hemmer W, Taylor SS (2000). Serine-53 at the tip of the glycine-rich loop of cAMP-dependent protein kinase: Role in catalysis, P-site specificity, and interaction with inhibitors. Biochemistry.

[pbio-0050017-b072] Johnson DA, Akamine P, Radzio-Andzelm E, Madhusudan M, Taylor SS (2001). Dynamics of cAMP-dependent protein kinase. Chem Rev.

[pbio-0050017-b073] Huse M, Kuriyan J (2002). The conformational plasticity of protein kinases. Cell.

